# Age-related changes to oscillatory dynamics during maintenance and retrieval in a relational memory task

**DOI:** 10.1371/journal.pone.0211851

**Published:** 2019-02-07

**Authors:** Renante Rondina II, Rosanna K. Olsen, Lingqian Li, Jed A. Meltzer, Jennifer D. Ryan

**Affiliations:** 1 Rotman Research Institute, Baycrest, Toronto, Ontario, Canada; 2 Department of Psychology, University of Toronto, Toronto, Ontario, Canada; 3 Department of Psychology, Ryerson University, Toronto, Ontario, Canada; 4 Department of Psychiatry, University of Toronto, Toronto, Ontario, Canada; Nathan S Kline Institute, UNITED STATES

## Abstract

In aging, structural and/or functional brain changes may precede changes in cognitive performance. We previously showed that despite having hippocampal volumes similar to those of younger adults, older adults showed oscillatory changes during the encoding phase of a short-delay visuospatial memory task that required spatial relations among objects to be bound across time (Rondina et al., 2016). The present work provides a complementary set of analyses to examine age-related changes in oscillatory activity during maintenance and retrieval of those spatial relations in order to provide a comprehensive examination of the neural dynamics that support memory function in aging. Participants were presented with three study objects sequentially. Following a delay (maintenance phase), the objects were re-presented simultaneously and participants had to determine whether the relative spatial relations among the objects had been maintained (retrieval phase). Older adults had similar task accuracy, but slower response times, compared to younger adults. Both groups showed a decrease in theta (2-7Hz), alpha (9-14Hz), and beta (15-30Hz) power during the maintenance phase. During the retrieval phase, younger adults showed theta and beta power increases that predicted greater task accuracy, whereas older adults showed a widespread decrease in each of the three frequency ranges that predicted longer response latencies. Older adults also showed distinct patterns of behaviour-related activity depending on whether the analysis was time-locked to the onset of the stimulus or to the onset of the response during the test phase. These findings suggest that older adults may experience declines in relational binding and/or comparison processes that are reflected in oscillatory changes prior to structural decline.

## Introduction

Aging is typically associated with changes in memory; in particular, aging disproportionately impairs *relational memory*, the memory for the relations between items, compared to memory for the individual items themselves. Older adults tend to perform less accurately and respond more slowly than younger adults on relational memory tasks, regardless of the type of relations (source, context, temporal order, location, item pairs), test materials (verbal, nonverbal), test formats (recognition, recall), and delay lengths (short, long) [1–5]. Aging is also associated with disproportionate atrophy to the hippocampus and adjacent structures in the medial temporal lobe [6–8], which are critical for the formation, or binding, of relational memories [9]. These structures have also been implicated in the subsequent *comparison* of externally presented relations to those that have been maintained in memory [10]. Functional magnetic resonance imaging (fMRI) studies have reported both over- and under-recruitment of the hippocampus in older adults during relational memory encoding [11–22]. Thus, age-related structural and functional changes in the hippocampus and broader MTL network may contribute to relational memory declines in older adults. However, when memory performance is preserved, older adults have been shown to recruit other neocortical areas during encoding [23,24] and retrieval [25]. Such patterns are suggestive of a compensatory response in older adults in which more, or different, neural activity is required to support successful task performance [26].

More recently, work has examined the extent to which aging is accompanied by changes in oscillatory activity across different phases of a memory task (e.g., encoding, maintenance). For example, in the encoding phase of a verbal [27] and visuospatial [28] working memory task, older adults showed more widespread alpha (~10Hz) and beta (~20Hz) power decreases than younger adults despite performing just as accurately as younger adults. During the maintenance phase of the verbal working memory task, older adults continued to show greater and more widespread alpha and beta power decreases than the younger adults [27]. Age-related differences in oscillatory dynamics have also been reported in visual working memory tasks in which older adults performed less accurately than younger adults. For instance, older adults showed weaker theta (~5Hz) coupling than younger adults during the maintenance phase [29], and older adults showed less consistent cue-evoked alpha phase resetting than younger adults during the retrieval period which predicted worse performance [30].

These age-related differences in oscillatory activity may reflect alternate cognitive processes engaged during task performance for older, compared to younger, adults. Theta frequency oscillations have been linked with successful memory formation [31–34], and theta power increases in younger adults during working memory maintenance have been well documented [35–38]. However, theta power decreases have also been reported during encoding [39,40] and maintenance [41,42]. In either case, age-related differences in theta frequency oscillations would reflect a deficiency in activating the same memory processes as younger adults. Indeed, previous studies have reported reduced theta power in older adults compared to younger adults, linked to deficits in memory [43] and cognitive mapping [44]. Alpha frequency oscillations have been linked with wakefulness and attention such that increases may reflect inhibition of task-irrelevant information [45–47]. Alpha and beta frequency oscillations are both negatively correlated with the BOLD signal in fMRI studies [48–51] such that power decreases in these frequency ranges may be viewed as a general-purpose indicator of neural activation. More recently, it has been proposed that alpha and beta power decreases in neocortical areas reflect a deeper level of processing (i.e., semantic vs. non-semantic) [33] and an increase in the richness of information being processed in a system [32]. Therefore, greater alpha and beta power decreases in older adults relative to younger adults may signal the use of different cognitive strategies (e.g., inhibition, or semantic strategies), and/or additional neural resources, by older adults to support task performance.

In our previous work [28,38], we employed a short-delay visuospatial memory task to examine the neural oscillations that support memory encoding, maintenance, and retrieval of the relative spatial relations among objects. On each trial, younger adults were presented with three novel objects, one at a time, with each object occupying a different spatial location on the screen. Following a delay, participants were re-presented with the three objects, simultaneously. All of the objects were moved in their absolute spatial positions, but participants were required to decide whether their relative positions had been maintained across the delay from the study to the test phase. The test phase, then, specifically required the participants to compare the externally presented information with information maintained in memory, and successful performance could not be achieved through recognition of a single item or through the detection of local luminance changes. Younger adults showed theta power increases in the hippocampus and medial prefrontal cortex as spatial relations were integrated across the study phase, and such binding-related theta power increases in the hippocampus were related to subsequent ability to detect a change in the relative spatial positions among the objects [38]. These findings provided evidence for a role for the hippocampus and theta oscillations in the binding of relational information across space and time, even across brief delays.

Our subsequent work [28] examined the extent to which aging was associated with changes in the recruitment of binding-related oscillatory activity in hippocampal and neocortical regions during the encoding phase. Using the same MEG paradigm and structural MRI [38], we contrasted hippocampal volume, behavioural performance, and patterns of oscillatory activity, and the relationships among them, between a group of younger adults from our previous study [38] and a group of healthy older adults. Although task accuracy and hippocampal volumes did not differ between younger and older adults, the older adults did not show the binding-related change in theta networks that had been previously observed in the younger adults. Instead, older adults showed an alpha power decrease in frontal, temporal, and parietal areas across the study phase that was not present in younger adults, as well as a beta power decrease in a more widespread network than younger adults. Age-related changes to oscillatory activity, despite hippocampal volumes that are similar to those of younger adults, suggests that, in aging, functional changes may occur prior to structural changes [28]. Further, these oscillatory differences between age groups may indicate that older adults encoded fundamentally different representations, or engaged different cognitive strategies, to support task performance.

The goal of the present study was to provide a complementary set of analyses to our prior work on encoding that would examine the extent to which age-related differences in oscillatory activity are observed during maintenance and retrieval. Further, we asked whether oscillatory activity during maintenance and/or retrieval would be associated with task performance. We predicted that older adults would show greater modulations of alpha and beta oscillations during the delay and retrieval phases relative to younger adults, and altered engagement of theta networks relative to younger adults during the retrieval phase [28,36]. To the best of our knowledge, no studies have investigated age-related differences in oscillatory activity during the maintenance or retrieval phase of a short-delay visuospatial memory task in the context of which task accuracy is comparable to younger adults. Furthermore, brain-behaviour relationships were examined to comprehensively understand the role that any age-related differences in oscillatory activity may have in task performance. The findings from the present study will build upon our previous work, and emerging research in the field of aging [36], to provide a comprehensive investigation into how memory is supported in aging.

## Methods

The participants, stimuli and design, procedures, and data acquisition were previously described in [28] and [38], and are repeated here for clarity.

### Participants

Sixteen younger adults (8 males; age: M = 24.8 years, SD = 3.5; education: M = 17.8 years, SD = 2.5) and 16 older adults (8 males; age: M = 65.9 years, SD = 6.6; education: M = 16.3 years, SD = 2.9) participated in the study. Participants were excluded if they scored lower than 26/30 on the Montreal Cognitive Assessment (MOCA; Damian et al., 2011) or had any psychiatric or neurological conditions. The groups did not differ in MOCA scores or years of education (all *p*s > 0.11). All participants were recruited from the Toronto community with normal neurological histories and normal or corrected-to-normal vision. The study was approved by the Research Ethics Board of Baycrest and the rights and privacy of the participants were observed. All participants gave informed consent before the experiment and received monetary compensation.

### Stimuli and design

**Fig 1:**
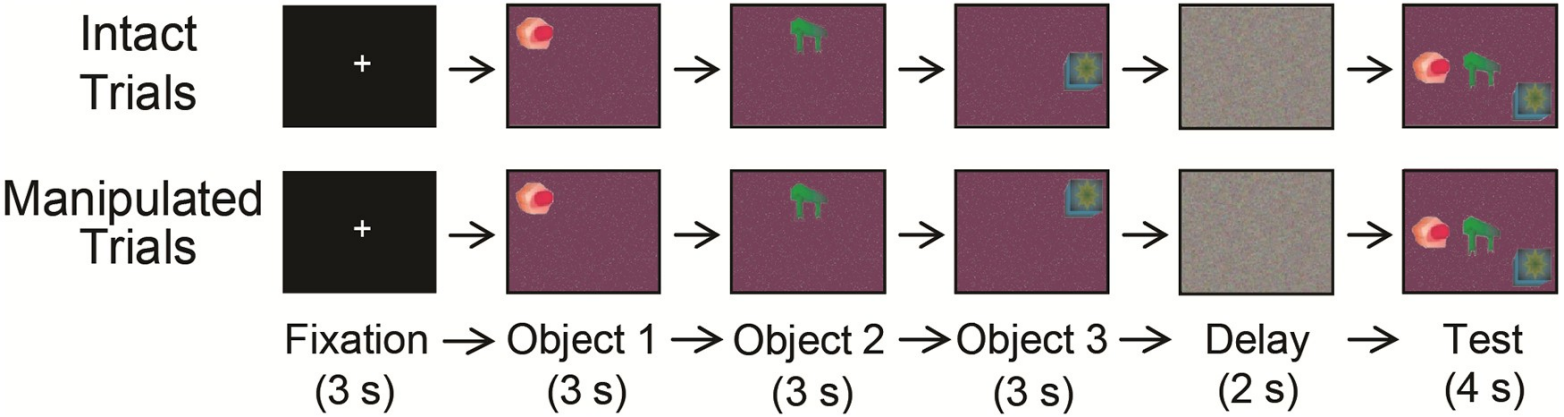
Task design. Participants were instructed to study the relative spatial locations of three objects (not drawn to scale) that are presented sequentially for 3 s each. After the study phase, a visual mask was presented for 2 s. At test, all three study objects were re-presented simultaneously in new absolute locations. The relative spatial locations between each object were either identical to those of the study phase (intact condition) or different (manipulated condition). Participants made their response during the 4-s test phase.

Stimuli were images composed of a patterned background with three abstract novel objects (non-nameable) superimposed, and were among the set used in prior work [38,52,53]. Images were grouped into sets that were distinguished by the patterned background and three objects that were unique to each trial (see Fig 1 for sample). Three study displays, each composed of a single object in a unique location, were sequentially presented to ensure that the relationships among stimuli had to be bound together across time. A mask image was used during the delay phase for each trial. The test displays consisted of all three studied objects presented simultaneously in new absolute spatial locations to ensure that low-level features, such as luminance changes at a given location, could not be used to aid performance. The relative spatial locations among the objects either remained intact (see Fig 1, top), or had been manipulated (see Fig 1, bottom).

### Procedure

Both younger and older adults completed the same experiment consisting of 204 trials Each set of images was preceded by a fixation cross that appeared at the centre of the screen for 3 s. Three study images were presented for 3 s each, followed by the presentation of a mask image for 2 s, and the test image for 4 s (see Fig 1). The relative spatial locations between objects in the test phase were intact relative to the study phase for half the trials and manipulated for the other half. Participants were instructed to indicate via a button press response whether the relative locations among the objects were maintained from study to test displays. Both younger and older adults delivered their responses during the test phase before the end of each trial. Participants were allowed to move their eyes freely during the task to explore the visual relationship among the objects. Participants were not instructed to maintain their fixation, as previous studies have shown that eye movements are functional for learning in both younger and older adults [54–57]. Specifically, restricting eye movements decreases subsequent memory performance compared to free viewing conditions [58,59], and has a larger consequence on memory for the spatial relations among objects compared to memory for intrinsic object features [60].

### Data acquisition

MEG recordings were performed in a magnetically shielded room at the Rotman Research Institute, Baycrest, using a 151-channel whole head first order gradiometer system (VSM-Med Tech Inc.) with detection coils uniformly spaced 31 mm apart on a helmet-shaped array. Participants sat in an upright position, and viewed the stimuli on a back projection screen that subtended approximately 31 degrees of visual angle when seated 30 inches from the screen. Participant’s head position within the MEG was determined at the start and end of each recording block using indicator coils placed on the nasion and bilateral preauricular points. These three fiducial points established a head-based Cartesian coordinate system for representation of the MEG data. The MEG system used presently for data collection did not track continuous head position in the scanner during the session. However, participants were excluded from the study if they moved more than 1 cm within each run. In order to co-register the brain activity with the individual anatomy, a structural MRI was also obtained for each participant using standard clinical procedures with a 1.5 T MRI system (Signa EXCITE HD 11.0; GE Healthcare Inc., Waukesha, WI) located at Sunnybrook Health Sciences Centre or with a 3 T MRI system (Siemens TIM Trio) located at Baycrest. All participants’ anatomical MRIs and MEG source data were spatially normalized using Advanced Normalization Tools (ANTs) to standard MNI_avg152T1 space to allow for group analysis of functional data. ANTs is a state-of-the-art medical image registration segmentation toolkit that uses a Symmetric Normalization (SyN) nonlinear registration algorithm to register brain image data to a common space for analysis [61].

### Data analysis

#### Hippocampal volumes

Possible age related differences in hippocampal volumes, as well the structure-function relationships during the pre-stimulus period and study phase, were examined in [28]. We repeat here our previously described procedures for volumetric analyses in order to examine possible age-related differences in the structure-function relationships during the delay phase and test phase. To conduct manual volumetric analyses of the hippocampal structures, we used the protocol described by [62]. Raw DICOM MRI images were first converted to NIFTI format. Next, all structural scans underwent signal-intensity normalization and non-uniformity correction using AFNI’s 3dUniformize, and transformation into standard stereotaxic space using the Talairach-Tournoux template prior to volume segmentation using AFNI’s @auto_tlrc [62–65]. Manual volumetric segmentation of the 1 mm isotropic MRI images was then conducted using FSLView by a single rater (L.L.) [66]. This software allowed viewing of volumes simultaneously in the sagittal, coronal and horizontal orientations of each slice. The total volumes (mm^3^) of the segmented structures was calculated using FSL’s fslstats function. For the purposes of the current investigation, the subregions of the hippocampus (head, body, and tail) were collapsed (summed) into a single region of interest for each subject (for left and right hemispheres, separately).

#### Source localization

The MEG data were analyzed in source space using Synthetic Aperture Magnetometry (SAM) [67–69] as implemented in CTF software (CTF, Port Coquitlam, BC, Canada). SAM is a beamformer technique that can be used to compute the full time course of virtual channels at selected individual locations, as in our previous work [38,70]. Analysis of virtual channel signals in source space also has two advantages beyond localization compared to analysis of sensor data: (1) the beamforming procedure attenuates extracranial artifacts such as blinks, eye movements, and muscle activity [71,72], and (2) source-space analysis compensates for differences in head shape and head position across participants, which strongly affect the propagation of electromagnetic activity from the brain to the sensors, which are fixed in the MEG helmet. Note that we did not reject trials based on blinks because the beamforming procedure effectively removes them from the virtual signals estimated for intracranial locations, with the possible exception of signals in the orbitofrontal cortex adjacent to the eye orbits [73]. Source waveforms were derived from locations throughout the brain in MNI space using the macroanatomical cortical parcellation of [74], consisting of 116 cortical/subcortical regions, including the cerebellum. Coordinates at the centre of each region were warped from MNI space to the individual brain, co-registered to the MEG head position, and beamforming weights for these locations were computed across a broad frequency range (1–100 Hz).

**Fig 2:**
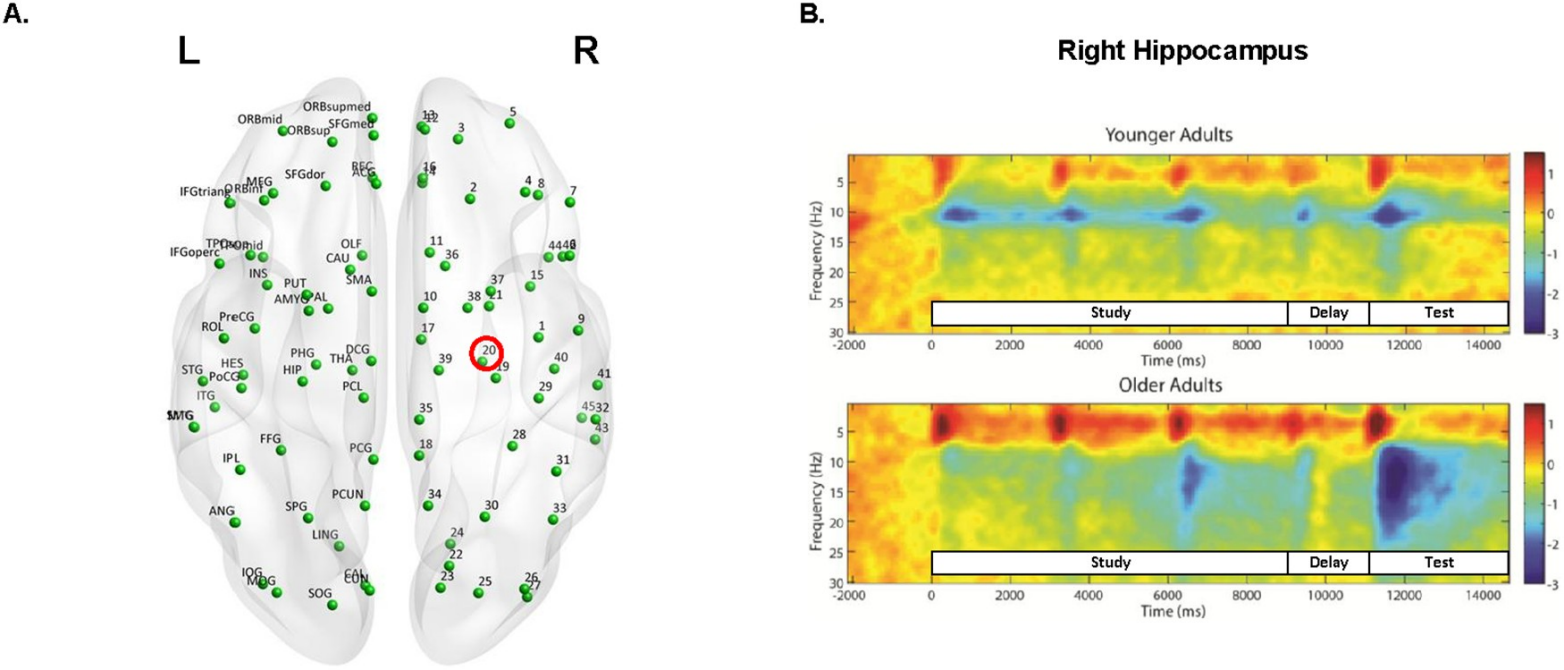
Virtual channel placements and right hippocampal time frequency plot. A: Axial view of the distribution of 90 virtual channel locations from the AAL atlas with region labels on the left (L) side and numbers representing homotopic regions on the right (R) side, and the right hippocampus outlined in red. B: Time frequency plots illustrating power changes for the younger and older adults for the right hippocampus with the time windows for the study phase (0 ms to 9000 ms), delay phase (9000 ms to 11000 ms), and test phase (11000 ms to 15000 ms) indicated.

To examine age-related differences in oscillatory activity throughout the brain, a time-frequency analysis on 90 of the 116 virtual channel locations was performed (see Fig 2A for distribution of sources, excluding the cerebellum). This first stage of analysis allowed for the delineation of the time and frequency windows in which oscillatory reactivity occurred in the task. Beamformer weights were computed using data from a window of -2.5 s to 15 s relative to the onset of the first study display, and submitted to a time-frequency analysis in EEGLAB [75], with a short-time Fourier transform (512-point overlapping Hanning windows, 200 windows per trial). Based on the observed reactivity, oscillatory power in the theta (2–7 Hz), alpha (9–14 Hz), and beta (15–30 Hz) frequency ranges during specified time windows were used as input for multivariate statistical analysis (see also 41). However, as oscillations below 4Hz have traditionally been considered to be part of the delta frequency range, an alternative analysis was conducted defining theta as 4–7 Hz that yielded predominantly similar results as our original analyses and are provided in supporting materials (see S1 Fig). Areas of difference between the two sets of results are highlighted in the Discussion section. Time windows are described in the following section, and were chosen to focus the analysis on sustained changes in oscillatory power, rather than the initial time-locked evoked response to the visual stimulus [76].

### Statistical analysis

#### Behaviour

Group differences in task accuracy and mean response time were reported previously [28] and are repeated here in Table 1.

**Table 1:** Hippocampal volumes and behaviour.

	Hippocampal Volumes (mm^3^)	Accuracy (%)	Mean Response Time (ms)
Younger Adults	7595.4 (940.4)	82.5 (9.2)	1543 (205)
Older Adults	7321.6 (812.9)	81.3 (10.7)	2042 (553)
	*t* < 1	*t* < 1	*t*_*(30)*_ = 3.38, *p* < 0.01

Values in parentheses show standard deviations.

#### Oscillatory activity during the delay phase

For the analysis of oscillatory activity during the delay phase, a time window of 0.75 s to 1.5 s after the onset of the delay phase was compared with the 0.75 s immediately preceding the onset of the study phase (i.e., the pre-stimulus interval). Oscillatory power from 90 virtual channels was submitted to a mean-centered partial least squares (PLS) analysis to assess differences between older and younger participants during the delay phase. PLS is a multivariate statistical technique [77–80] that allows simultaneous examination of conditions and age-related group effects in multi-dimensional MEG data [81]. PLS uses singular value decomposition (SVD) to identify multivariate patterns so that traditional multiple comparison correction is not necessary. Resampling methods are then used to identify virtual channels where the pattern is reliable.

For the maintenance phase, the conditions were the pre-stimulus period (-0.75 s to 0 s) versus the delay period (0.75 s to 1.5 s), and the dimensions of the MEG data included spatial locations (90 virtual channels) and frequency band (theta, alpha, and gamma). Data submitted to the mean-centered PLS was organized with oscillatory power at each virtual channel location within each frequency range in the columns, and the participants within conditions within groups in the rows. The data matrix was mean-centered and decomposed with SVD, which produces a set of mutually orthogonal latent variables (LVs) that account for the greatest possible variance in data across conditions of interest. Each LV consists of: (i) design salience (or design LV), which shows the contrast between conditions and/or groups; (ii) brain saliences that indicate the distribution of oscillatory brain activity identified by the design LV; and (iii) a singular value that represents the covariance between the brain salience and the design LV. Design LVs are expressed in terms of “brain scores”, which describes the degree to which each participant expresses the observed pattern of brain activity.

Statistical assessment in PLS consisted of two independent resampling methods: permutations and bootstrap estimations. Permutation tests evaluated the probability that the singular values (covariances) associated with the optimal contrast would be obtained with any other random permutation of condition labels [78]. In this non-parametric test, the participants’ data were randomly reassigned to conditions and a new set of LVs was calculated for each reordering. At every permutation, the singular value obtained was compared to the singular value from the original data, and was assigned a probability value based on the number of times the singular value from the permuted data exceeded the original value. Bootstrapping determined the reliability of saliences (or weights) for distributed power changes in virtual channel locations [78,82]. Bootstrap estimation involved randomly sampling subjects with replacement while keeping assignment of experimental conditions fixed for all observations and then computing the standard error of the saliences for each virtual channel location and frequency range [79]. Virtual channel locations were thresholded at 95 percent confidence bounds derived from the bootstrap distribution. These two resampling techniques provided complementary information about the statistical strength of the task contrast and brain-behaviour relationship, and their reliability across participants. Statistical evaluation of oscillatory effects was tested using 1000 permutations and 1000 bootstrap iterations. Results were considered significant at alpha = 0.05.

#### Structure-function and brain-behaviour relationships during the delay phase

Oscillatory power changes during the delay phase from 90 virtual channel locations (the delay phase minus the pre-stimulus period) were submitted to a behavioural PLS with total recognition accuracy, mean response time to correct trials, and hippocampal volumes as predictors. Behavioural PLS [77–80] was used to assess brain-behaviour correlations simultaneously for all conditions and groups. The input to the behavioural PLS was a cross-correlation matrix created from the oscillatory power of each participant and their behavioural measures on the task. This cross-correlation matrix was decomposed using SVD, and statistical assessment was evaluated with 1000 permutations and 1000 bootstrap iterations, as described above.

#### Oscillatory activity during the test phase

In our previous study [38], we analyzed a time window of 0.25s to 0.75 ms to measure early oscillatory responses towards the test stimuli in younger adults. However, preliminary inspection of the virtual channel data suggested that age-related differences persisted throughout the duration of the test phase. Therefore, an independent mean-centered PLS was used to assess differences in stimulus-locked oscillatory activity during the test phase between older and younger participants by comparing a time window of 0.25 s to 2.5 s after the onset of the test stimuli to a similar time window of 0.25 s to 2.5 s after the onset of the first study displays. In this way, the test phase is compared to a period in which memory demands are minimal and differences in bottom-up visual information between conditions are reduced. An alternative analysis comparing the test phase to the pre-stimulus interval yielded similar results and is provided in the supporting materials (see S2 to S4 Figs). Response-locked neural activity was assessed by comparing the oscillatory power from the first study display (0.25 s to 1.25 s) to that from a time window immediately preceding a response (-1.0 s to 0 s relative to a button press). To avoid capturing activity that occurred during the delay period, this analysis only included trials in which participants responded more than 1 s following the onset of the test stimulus. Younger and older adults did not differ in the number of trials included in this analysis (younger adults: mean = 140.25, range 112–167; older adults: mean = 152.44, range: 75–187), *t*_(30)_ = 1.49, *p* > 0.145). Only correct trials were analyzed. The data matrix submitted to PLS was organized similarly as described above, mean-centered and decomposed using SVD, and the statistical assessment was evaluated with 1000 permutations and 1000 bootstrap iterations. Differences in test phase activity between intact and manipulated trials for the stimulus- and the response-locked analyses were also examined, but returned no significant effects, and so are not reported here.

#### Structure-function and brain-behaviour relationships during the test phase

Oscillatory power changes during the test phase from 90 virtual channel locations (the test phase minus the first study display) were submitted to a behavioural PLS with total recognition accuracy, mean response time to correct trials, and hippocampal volumes. The input to the behavioural PLS was a cross-correlation matrix as described above, decomposed using SVD, and the statistical assessment was evaluated with 1000 permutations and 1000 bootstrap iterations.

## Results

### Hippocampal volumes and behavioural results

Hippocampal volumes and behavioural performance (accuracy, response time) are reported here in Table 1 as in [28]. There were no significant age differences in hippocampal volumes or task accuracy. Older adults responded more slowly (*M* = 2042 ms; *SD* = 553 ms) than younger adults (*M* = 1543 ms; *SD* = 205 ms), *F*_(1,30)_ = 11.77, *p* < 0.001.

To ensure that age differences in response times were not due to issues of fatigue in the older adults, we examined whether older adults had disproportionately longer RTs for trials that appeared later in the study. In fact, both younger and older adults had *faster* response latencies on the last 102 trials (*M* = 1766 ms; *SD* = 316 ms) than on the first 102 trials (*M* = 1900 ms; *SD* = 353 ms), *F*_(30)_ = 19.614, *p* < 0.001. There was no interaction with age. Therefore, the present findings, including any observed age-related changes in oscillatory activity, are not likely due to fatigue.

Note that, as previously reported [28], one older adult’s accuracy was within 2.5 SD of the mean for the older adult age group, but this participant’s response time was outside of the 2.5 SD range. Removing this older adult did not change any of the results with respect to average hippocampal volumes, accuracy, oscillatory activity, or the relationship among hippocampal volumes, accuracy, and oscillatory activity; therefore this participant was included in these analyses. However, this participant was excluded from analyses that examined the brain-behaviour relationship between response-locked oscillatory behaviour and response times.

### Head movement

To rule out the possibility that greater head movement in older adults may contribute to the effects reported here, we submitted absolute changes in fiducial marker positions between runs to a three-way mixed design ANOVA with age as a between subject variable (younger adults, older adults), and fiducials (nasion, left ear, right ear) and axis (x,y,z) as within subject variables. There was a significant three-way interaction *F*_(120)_ = 2.88, *p* = 0.03, which revealed that younger adults showed greater movement between runs in the nasion fiducial along the z-axis (0.631) than did the older adults (0.255 cm), *t*_(30)_ = 2.23, *p* = 0.03.

### Recruitment of oscillatory activity during the delay phase

**Fig 3:**
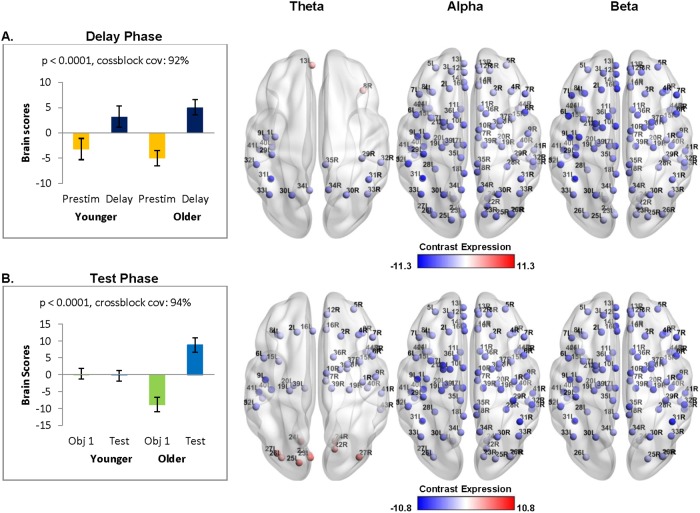
Oscillatory power changes across groups for the delay phase (A) and test phase (B). Error bars signify confidence bounds (95%) obtained from the bootstrap distribution. Distribution of virtual channels that positively (red) and negatively (blue) express the contrast are shown on the bottom of each analysis. A: Both groups showed a widespread power decrease from the pre-stimulus period to the delay phase across all frequency ranges. B: Only older adults showed a widespread power decrease from the first study display to the test phase in all frequency ranges.

Neural activity during the delay phase was assessed by comparing the oscillatory power from the pre-stimulus interval (-0.25 s to 0 s) to that from the delay phase (0.25 s to 1.5 s) (see Fig 2B for the time windows in the time frequency plot). This compares a time period during which participants ostensibly are maintaining online an already-formed representation of the relative spatial positions among the objects (delay phase) to a time period with a similar amount of visual information presented (i.e., no objects on the screen), but there has not yet been an opportunity for relational binding or maintenance of such a bound representation (pre-stimulus interval). Changes in theta, alpha, and beta power were examined for each group and group differences were assessed. Mean-centered PLS identified a significant pattern of brain activity that accounted for 92% of the covariance in the data, and captured differences between the pre-stimulus interval and the delay phase (*p* < 0.0001) but did not differ between groups. The red nodes indicate a positive theta modulation (delay phase > pre-stimulus interval) in frontal and temporal areas, while the blue nodes indicate widespread negative modulations (delay phase < pre-stimulus interval) in all three frequency ranges in frontal, temporal, and parietal areas (Fig 3A).

### Recruitment of oscillatory activity during the test phase

Neural activity during the test phase was assessed by comparing the oscillatory power from the first study display (0.25 s to 2.5 s) to that from the test phase (0.25 to 2.5s) (see Fig 2B for the time windows in the time frequency plot). This compares a time period in which participants were presumably engaged in retrieval and comparison processes (test phase), to a time period in which visual information was also presented, but binding demands were minimal as only one object had been presented (first study display) (see S2 to S4 Figs for alternative analyses comparing test phase activity to pre-stimulus baseline). Changes in theta, alpha, and beta power were examined for each group and group differences were assessed. Comparisons between intact versus manipulated trials during the test phase revealed no significant effects; thus, the presented results compare oscillatory activity from all trials in the test phase to all trials from the first study display of the encoding phase.

Mean-centered PLS identified a significant pattern of brain activity that accounted for 94% of the covariance in the data and captured group differences between the first study display and the test phase (*p* < 0.0001). The results in Fig 3B demonstrate that oscillatory power changes during the test phase are reliable for older adults only (confidence intervals cross zero for the younger adults). The red nodes indicate a positive theta modulation (test phase > first study display) in occipital areas, while the blue nodes indicate a widespread negative theta (first study display > test phase) modulation in frontal, temporal, and parietal areas. Older adults also showed negative modulations of alpha and beta frequencies across a widely distributed network.

**Fig 4:**
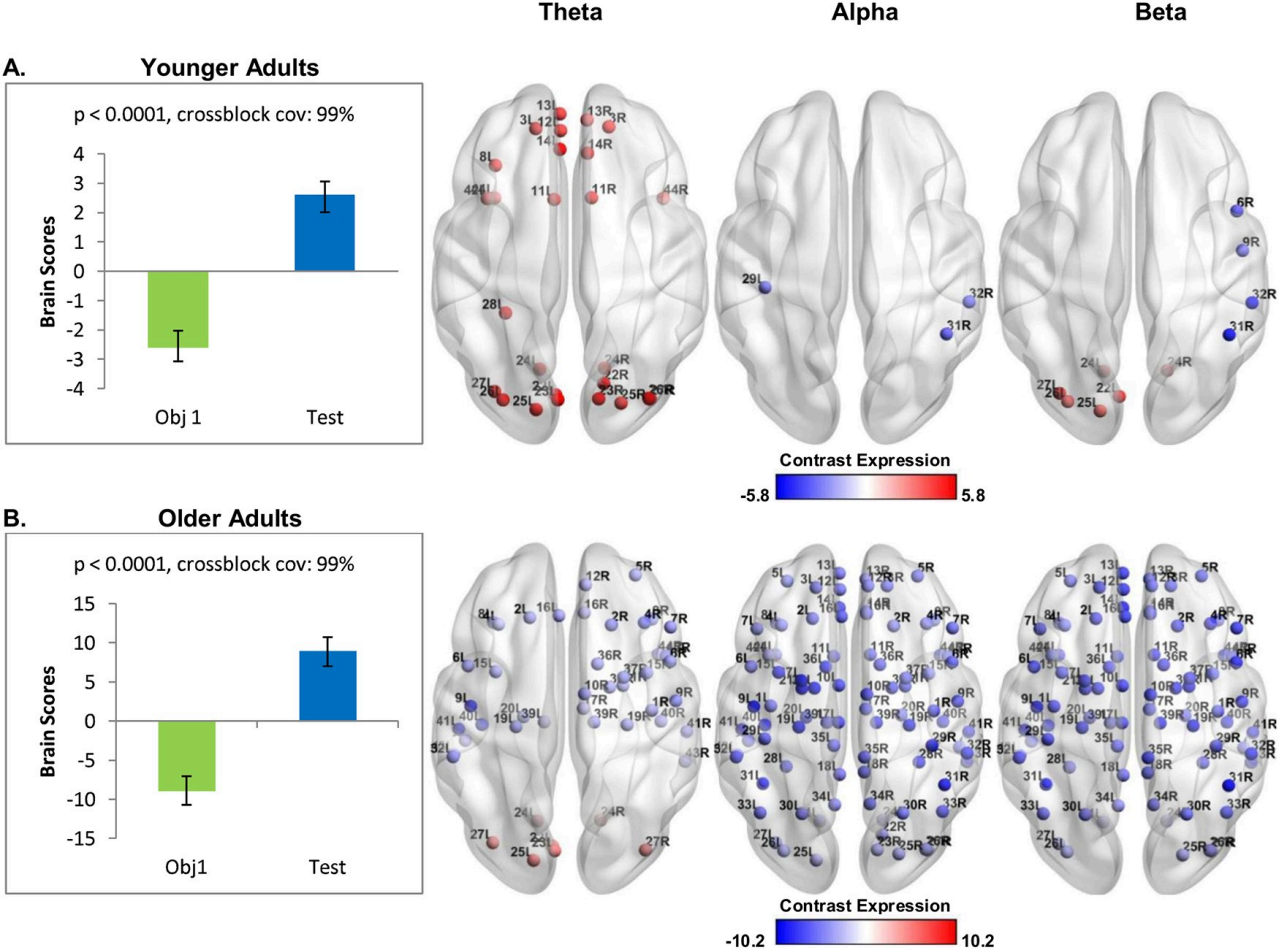
Oscillatory changes during the test phase in the younger adults (A) and the older adults (B). Error bars signify confidence bounds (95%) obtained from the bootstrap distribution. Distribution of virtual channels that positively (red) and negatively (blue) express the contrast are shown on the bottom. A: Younger adults showed a frontal and occipital theta increase, temporal alpha decrease, occipital beta increase, and frontotemporal beta decrease from the first study display to the test phase. B: Older adults showed an occipital theta increase, and widespread power decreases across all frequencies.

It is possible that the effects observed in older adults (Fig 3B) may have overwhelmed a significant, albeit smaller effect in the younger adults. To explore this, a separate mean-centered PLS was performed on the younger participants only. This identified a significant pattern of activity that accounted for 99% of the covariance in the data (*p* < 0.0001). The results in Fig 4A demonstrate that during the test phase, positive modulations (test phase > first study display) were observed for younger adults in theta in frontal, temporal, and occipital areas, and beta in occipital areas. The blue nodes indicate that a negative modulation (first study display > test phase) was observed in alpha in temporal regions, and in beta in frontal and parietal areas.

A separate mean-centered PLS was also performed on the older participants only. This identified a significant pattern of activity that accounted for 99% of the covariance in the data (*p* < 0.0001). The results in Fig 4B show that during the test phase, positive modulations (test phase > first study display) were observed for older adults in theta in occipital areas. Widespread negative modulations (first study display > test phase) were observed in alpha and beta, and fronto-temporal theta.

#### Structure-function and brain-behaviour relationships during the test phase

**Fig 5:**
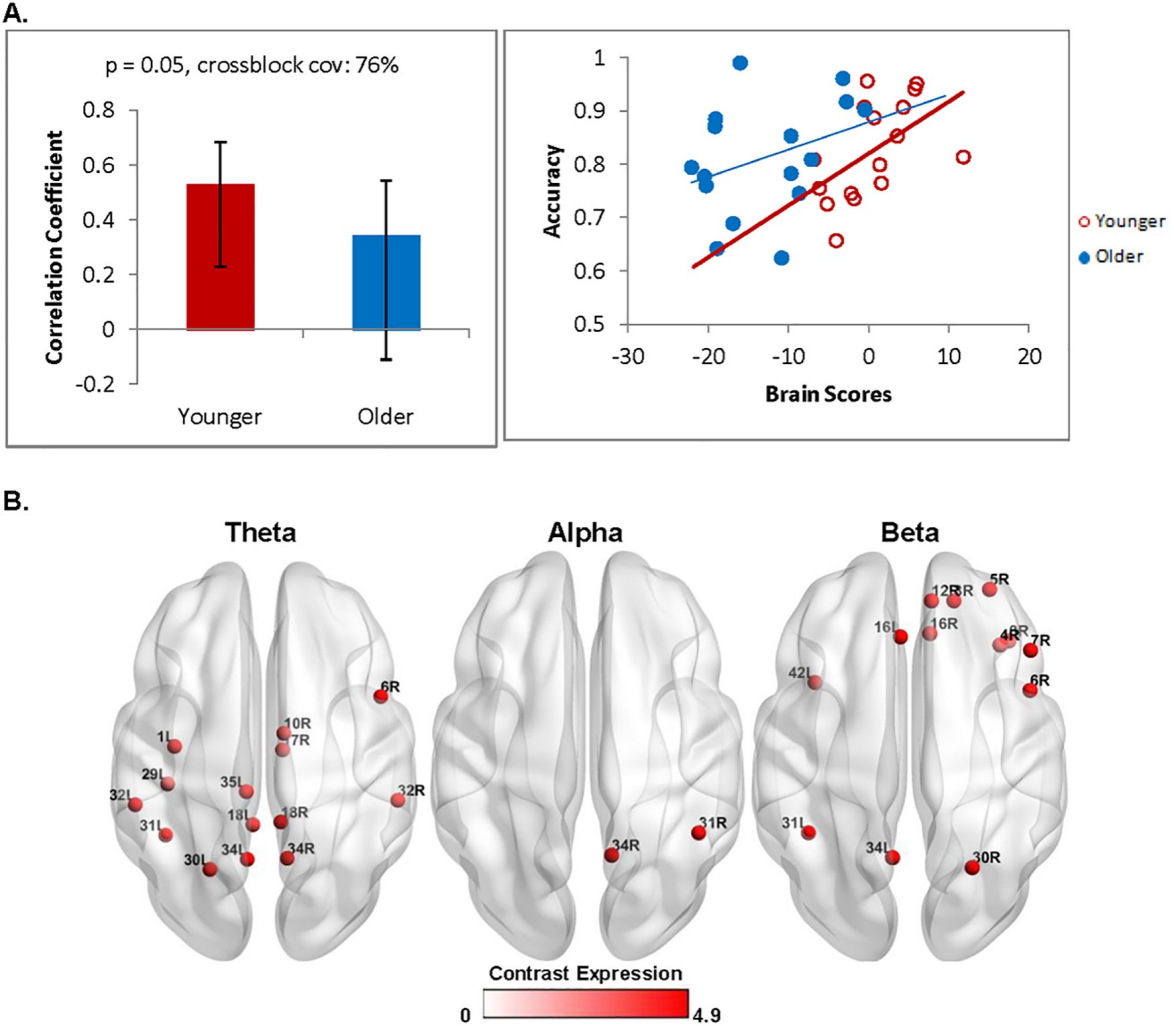
Stimulus-locked test phase brain-behaviour differences accuracy across groups. (A, left) Contrast shows group differences that are reliable for younger adults only. (A, right) The scatterplot recapitulates the effects by showing the distribution across individuals across groups with no obvious outliers present. (B) Distribution of virtual channels that positively express the contrast is shown for the three frequency bands of interest. Younger adults showed theta, alpha, and beta power increases that predicted higher task accuracy.

To comprehensively understand the impact of age-related changes in oscillatory activity, a behavioural PLS was conducted to examine the relationship between oscillatory activity and behaviour (see S1 Fig for alternative analyses defining theta as 4-7Hz). There was a significant relationship between oscillatory activity in the test phase (minus the first study display) and accuracy (*p* = 0.05, crossblock covariance: 76%) that was reliable in younger, but not older adults (confidence intervals cross zero bounds, Fig 5A). As shown in Fig 5B, the red nodes indicate a positive relationship between accuracy and theta, alpha, and beta power changes (*r* = 0.53) for younger adults in a number of frontal, temporal, and parietal virtual channels.

**Fig 6:**
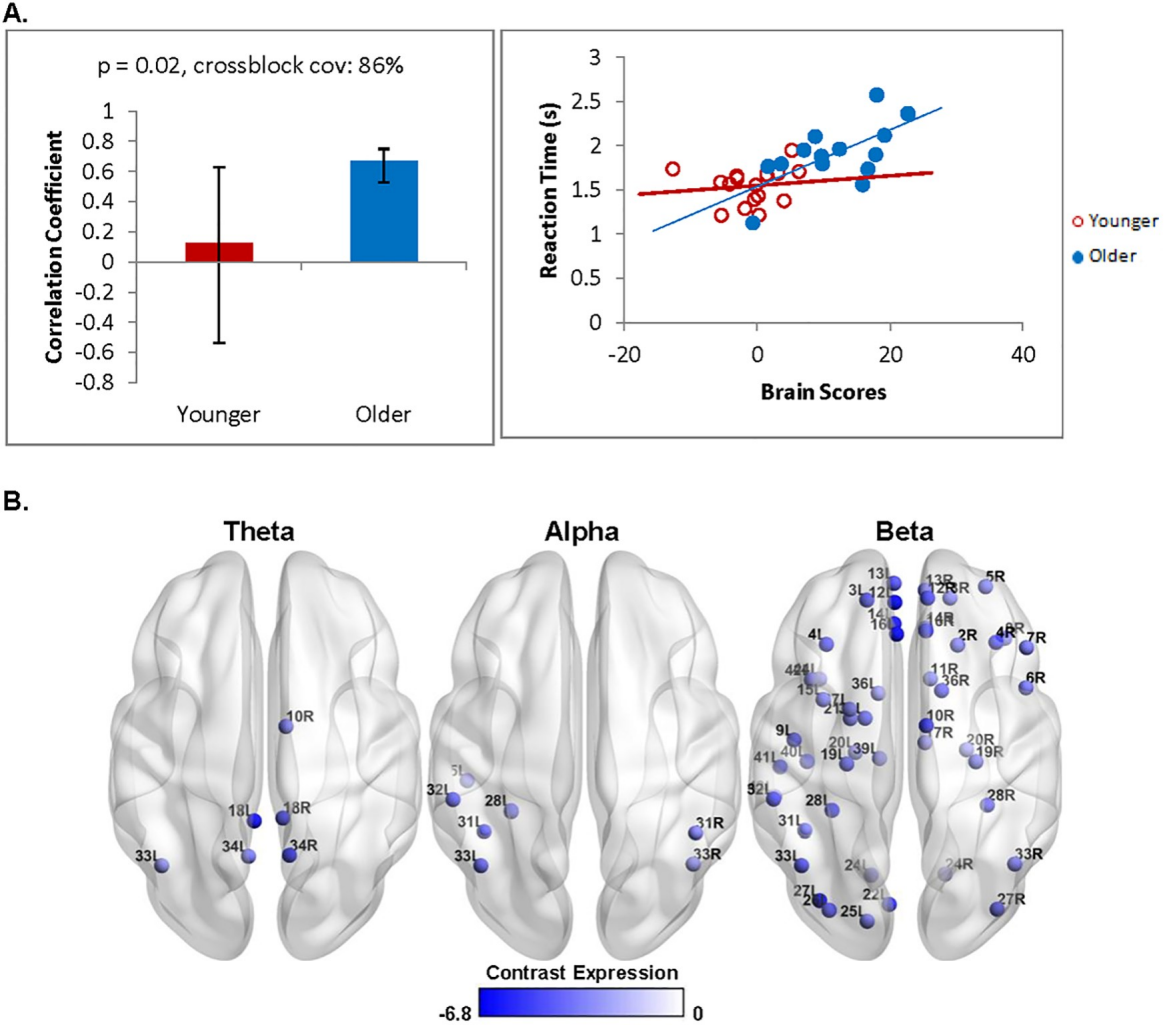
Stimulus-locked test phase brain-behaviour differences with response latencies across groups. (A, left) Contrast shows group differences that are reliable for older adults only i.e. confidence bounds computed from bootstrap estimation are stable. (A, right) The scatterplot recapitulates the effects by showing the distribution across individuals across groups with no obvious outliers present. (B) Distribution of virtual channels that negatively express the contrast is shown for the three frequency bands of interest. Older adults showed a theta, alpha, and beta decrease that predicted longer response latencies.

By contrast, there was a significant relationship between oscillatory activity in the test phase and response times (*p* = 0.05) that was reliable in older adults but not younger adults. This was still significant after removing the older adult with a mean response time outside of the normal range for his/her age group crossblock covariance: 86%; Fig 6A). As shown in Fig 6B, the blue nodes indicate a negative relationship between mean response time and beta power in frontal, temporal, parietal, and occipital areas (*r* = 0.61) such that longer response times were linked with greater power decreases. A similar negative relationship was also observed with theta power in frontal and parietal areas, as well as alpha power in parietal areas.

#### Response-locked neural oscillations

**Fig 7:**
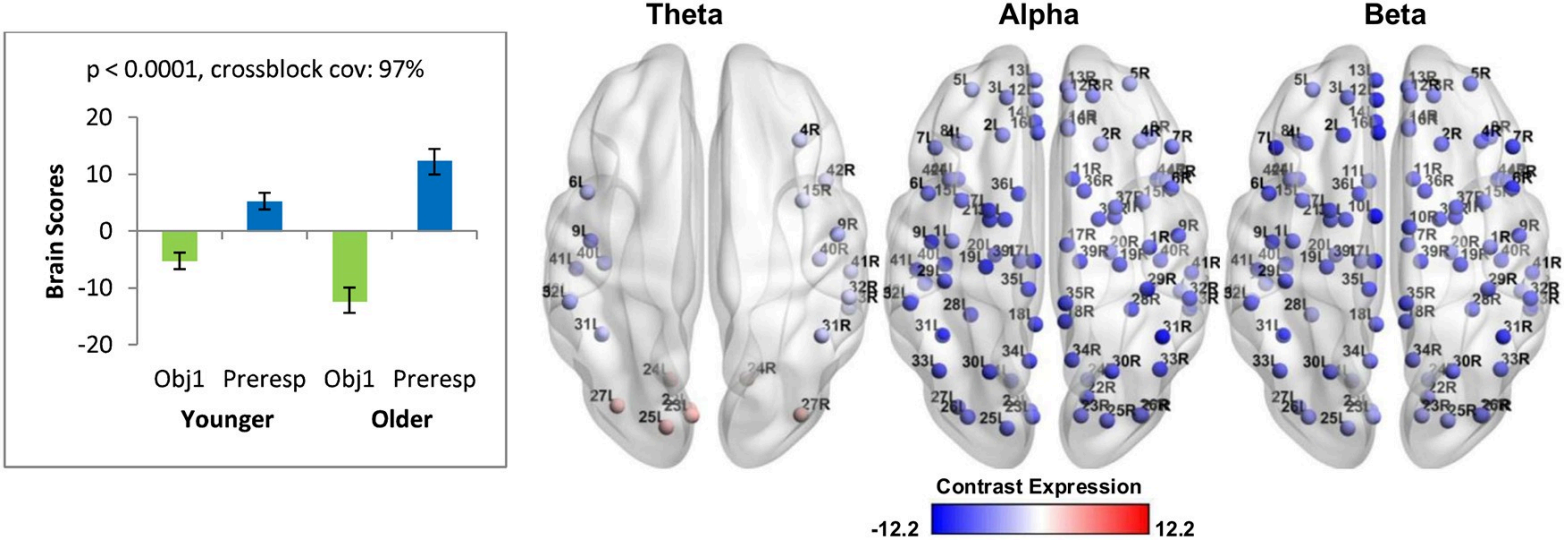
Response-locked oscillatory power changes (first study display vs. test phase) across groups were captured by a single latent variable. Error bars signify confidence bounds (95%) obtained from the bootstrap distribution. Distribution of virtual channels that positively (red) and negatively (blue) express the contrast are shown on the right for three frequency bands of interest (theta, alpha, and beta). Older adults express the pattern more reliably than younger adults across all frequency ranges.

To further explore the age-related power differences in the test phase, oscillatory activity preceding a behavioural response was examined. Trials in which participants responded faster than 1 second were excluded to avoid capturing activity from the delay phase. Mean-centered PLS identified a significant pattern of neural oscillations that differed between the first study display and the period immediately preceding a behavioural response and accounted for 97% of the covariance in the data (*p* < 0.0001). The results in Fig 7 demonstrate that the pattern of oscillatory activity prior to the response in the test phase was expressed more strongly by older than younger adults. The red nodes indicate a positive theta modulation (test phase > first study display) for both groups in occipital areas, while the blue nodes indicate a negative theta modulation (first study display > test phase) for both groups in frontal, temporal, and parietal areas. Widespread negative modulations of alpha and beta frequencies were also observed across a widely distributed network. Similar patterns of oscillatory activity was therefore observed between younger and older adults, and this pattern of activity differed from that observed for the stimulus-locked analyses that examined the relationship with response time.

#### Response-locked structure-function and brain-behaviour relationships

**Fig 8:**
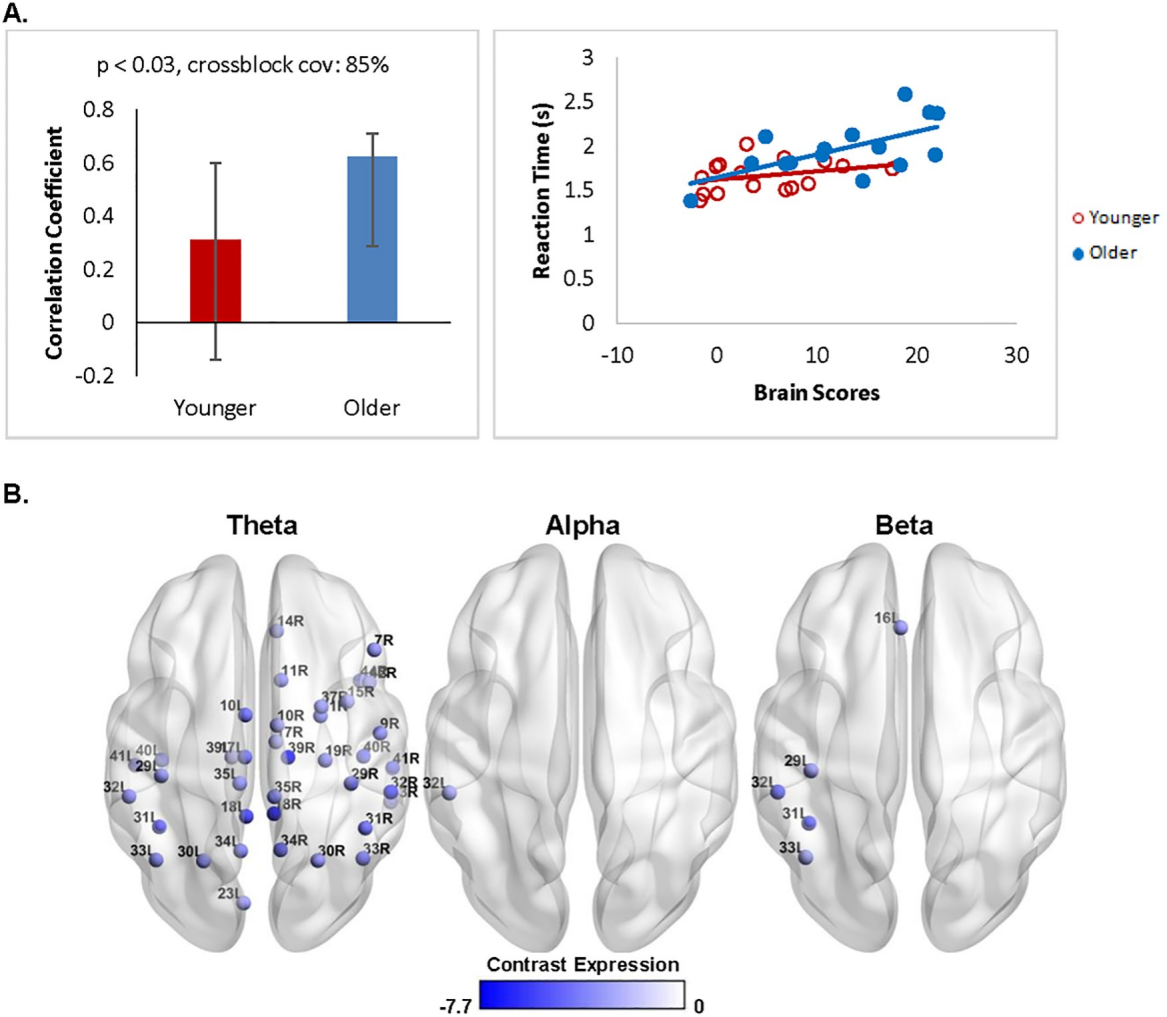
Response-locked brain-behaviour differences across groups. (A, left) Contrast shows group differences that are reliable for older adults only (A, right) The scatterplot shows the distribution of individuals from each age group. (B) Distribution of virtual channels that positively (red colors) and negatively (blue colors) express the contrast are shown for three frequency bands of interest (theta, alpha, and beta). Older adults showed a power decrease in all three frequency ranges that predicted longer response times.

To further probe the age-related oscillatory power changes in the response-locked analysis, a behavioural PLS was conducted to examine the relationship between oscillatory activity and behaviour. There was a marginally significant relationship between neural oscillations preceding the behavioural response and mean response times in older adults, but not younger adults (*p* = 0.087, crossblock covariance: 83%). This relationship was significant after removing the older adult with a mean response time outside of the normal range for his/her age group (*p* = 0.030, crossblock covariance: 85%; Fig 8A). As shown in Fig 8B, the blue nodes indicate a negative relationship between mean response time and theta power for older adults in the frontal, temporal, parietal, and occipital areas (*r* = 0.63) such that longer response times were linked with greater power decreases. A similar negative relationship was also observed with alpha power in the left supramarginal gyrus, and beta power in frontal and parietal areas.

## Discussion

The present study examined the differential recruitment of oscillatory activity in younger versus older adults across the maintenance and retrieval phases of a short-delay visuospatial memory task, in which the relative positions among objects had to be maintained across space and time. Behavioral performance (accuracy) was comparable between younger and older adults, and there was no evidence for age-related atrophy in the hippocampus [28]. During the maintenance phase, younger and older adults recruited similar, widespread, oscillatory networks across the three frequency ranges (theta, alpha, beta). However, compared to younger adults, older adults recruited different oscillatory networks at retrieval. Whereas younger adults showed an increase across the three frequency ranges during retrieval that predicted task accuracy, older adults showed a decrease across the three frequency ranges that predicted longer response latencies. The present study builds upon our prior work to suggest that, in advance of major behavioural and hippocampal volume changes, aging is accompanied by large changes in oscillatory reactivity during retrieval as well as in encoding [28]. These age-related changes in oscillatory responses may be indicative of declining ability to construct and subsequently use memory representations that contain information regarding the relations among items, and/or a change in the nature of information and cognitive strategy that is used by older adults to support performance [1,2].

Younger and older adults showed similar patterns of widespread power decreases across all three frequency bands during the maintenance period. Power decreases were strongest among frontal and parietal areas, including the left inferior parietal lobule, which is associated with language [83] and spatial cognition [84], as well as the left caudate nucleus, which has been implicated in working memory updating [85]. This stands in contrast with prior work that has found significant age differences during memory maintenance. However, such prior studies have either used verbal rather than visuospatial stimuli, or have examined oscillatory responses in the context of an age-related impairment in behaviour [27,29,86]. Age-related differences in oscillatory activity during the maintenance phase of a short-delay memory task may be more readily observable when the relational binding demands are high and/or when subsequent performance is less accurate than that of younger adults. Future research could parametrically manipulate the relational load of the task (i.e., the number of relative spatial relations among objects to be remembered) to encourage group differences in performance, and determine if age-related differences in oscillatory activity during the maintenance phase are predictive of subsequent task performance.

At retrieval, age-related changes were observed in the patterns of oscillatory activity. When oscillatory analyses were time-locked to the onset of the test stimulus and compared to the first study display, younger adults showed theta power increases that were maximal in frontal and temporal areas, including the left fusiform gyrus, which is associated with object perception [87] and object recognition memory [88]. Younger adults also showed alpha and beta power decreases in frontal and parietal areas including the right inferior parietal lobule. In contrast, older adults showed widespread power decreases across all three frequency bands regardless of whether the first study display or pre-stimulus interval was used as the comparison condition, and this was maximal in the right inferior parietal lobule, and included bilateral hippocampus, which is suggestive of altered hippocampal recruitment. This is consistent with other studies reporting theta increases in younger adults at retrieval [31,38], and theta and beta decreases in older adults [36,43]. Furthermore, task accuracy in younger adults was predicted by power increases across all three frequency ranges that was strongest in frontal areas, as well as bilateral superior and inferior parietal lobules, and theta and beta power increases in bilateral precuneus, suggestive of spatial and object memory processes [84]. In contrast, longer response times in older adults were predicted by stimulus-locked alpha and beta power decreases in the fusiform gyrus and inferior parietal lobule, as well as theta power decreases in the precuneus. Theta power increases, specifically in frontal areas, have been associated with successful memory retrieval [76,89–91]. In contrast, alpha and beta oscillations are known to be associated with attention and processing speed [45–47,92,93]. This suggests that older adults with comparable hippocampal volumes and accuracy rates to those of younger adults nonetheless recruited different oscillatory networks. Together with age-related slowing of response latencies, these findings point toward a more generalized decline in the speed of processing with aging.

Older adults also showed distinct patterns of behaviour-related activity depending on whether the analysis was time-locked to the onset of the stimulus or to the onset of the response during the test phase. The stimulus-locked analysis examined a longer time window through the test phase (250 to 2500ms), which may have captured sustained neural activity supporting task performance. Indeed, previous studies have found that beta power decreases in older adults were associated with lower performance in a visual attention task [92,93]. Stimulus-locked oscillatory patterns were dominated by beta power decreases that included bilateral fusiform and the left inferior parietal lobule, suggesting that longer response latencies may be due to a decline in visuospatial attention processes [84,87]. In contrast, the response-locked analysis may have captured later stages of memory retrieval (e.g., comparing the cue stimulus to previously encoded information) revealed by theta power decreases that included bilateral precuneus and the right hippocampus. Critically, this response-locked brain-behaviour relationship persisted regardless of whether the first study display or the pre-stimulus interval was used as a comparison condition (see S4 Fig). Decreased recruitment of medial temporal lobe structures may indicate a declining ability to recruit relational comparison processes with age [94].

Age-related changes were observed at retrieval, regardless of whether the oscillatory activity was compared to the first study display (Figs 4 to 8) or the pre-stimulus interval (S2 to S4 Figs). When we compared the test phase to the pre-stimulus period, both younger and older adults showed power decreases in all three frequency ranges (see S2 Fig). This is consistent with our previous study [28], in which younger adults showed a theta power decrease from the pre-stimulus interval to the study phase. However, although there was a general theta power decrease from baseline to task, younger adults showed a theta power increase from the first study display to the test phase. In contrast, older adults showed power decreases in all three frequency ranges that predicted longer response latencies, regardless of whether test phase activity was compared to the first study display or the pre-stimulus period.

In the present study, we defined theta as 2-7Hz, which was based on a visual inspection of the task-based low-frequency responsivity present in the time-frequency plots (see Fig 2; also see [28,38]. This is consistent with previous studies from other groups that have defined theta with a lower bound of ~2Hz [95]. While the frequency range of hippocampal theta has traditionally been defined as ~ 4-8Hz in non-human animals, there has also been work that suggests that human hippocampal theta may be more appropriately defined at a lower range of 1-4Hz [96], although frequencies below 4Hz may also be considered as part of the delta frequency range. We re-analyzed the data redefining theta as 4-7Hz. The results of these new analyses were consistent with our original findings defining theta as 2-7Hz, except for the behavioural PLS with stimulus-locked test-phase activity and performance accuracy (see S1 Fig). In the original analyses (theta as 2-7Hz), we found a significant relationship such that power increases in all frequency ranges predicted higher accuracy in younger adults (see Fig 5). However, in the alternative analysis (theta as 4-7Hz), this relationship was now reliable in both younger and older adults (confidence intervals do not cross zero). The brain-behavior relationship for task accuracy is therefore observed in older adults as in younger adults when the theta range is restricted to the high end. When considered alongside the observed age-related differences in the pattern of oscillatory responses for the test phase (Fig 4), as well as brain-behavior relationship for response times, these findings suggest that older adults recruit additional, rather than different, networks than younger adults. The observed shift to higher frequency ranges, observed here in the theta range, is consistent prior work on aging [97,98]. This may reflect the engagement of alternate memory processes by older adults, or the same relational memory processes may be engaged in older as in younger adults, but such processes may be supported by higher theta frequencies with aging.

The behavioural findings reported in the present study stand in contrast to numerous findings in the aging literature. Older adults typically perform less accurately than younger adults during recall and recognition tasks regardless of delay length [1,2], as well as tasks of spatial memory [99] and working memory [100]. In contrast, our older adults performed just as accurately as younger adults in a short-delay visuospatial memory task, which may be due to a low level of difficulty, preserved hippocampal volumes in the older adults, or because our older adults were younger than those in other typical aging studies (age range: 55 to 77 years of age). Nonetheless, the age-related differences in oscillatory activity at encoding and retrieval suggest that older adults may achieve similar levels of accuracy as the younger adults by engaging alternate oscillatory networks to compensate for declining function. Older adults also performed more slowly than the younger adults, which is consistent with aging studies across a range of tasks [101], including signal detection tasks [102] and visual choice reaction time tasks [103]. The present study adds to the literature by showing that, in the context of a short-delay relational memory task, longer response latencies in older adults may be due to a deficiency in visuospatial attention and/or relational comparison processes.

A limitation of the present study is that measures of eye movements were not collected during the experiment. We have shown in our previous studies that the amount of visual exploration in younger adults is related to hippocampal BOLD responses [104], and that although older adults make more saccades than younger adults, the relationship between eye movements and hippocampal BOLD responses is weaker in older adults than in younger adults [105], suggesting that older adults may increase visual exploration in order to up-regulate their memory system. Altogether, this suggests that the impact of eye movements on neural activity reflects learning and memory that occur during the course of the task. However, as participants freely explored the visual relationship among the objects, noise could have been introduced into the data due to artifact from the muscle movements of the eyes. Future studies could conduct simultaneous eyetracking with MEG recordings to examine the relationship between free viewing and the neural responses underlying visuospatial relational memory, and to account for any evidence of eye movement-related noise in the data.

The present study builds upon our previous work to provide a comprehensive characterization of the differential recruitment of oscillatory activity in younger and older adults across all three phases of memory: encoding, maintenance, and retrieval. In our prior work, older adults with intact hippocampal volumes and performance accuracy showed deficient theta recruitment and greater alpha/beta engagement at encoding compared to younger adults [28]. During retrieval, these older adults continued to show deficient theta recruitment, and the widespread power decreases across all frequency bands predicted slower reaction times. The age-related differences in oscillatory activity at retrieval may indicate a declining ability to engage in comparison of the maintained representation to the externally presented stimulus. Alternatively, the aging effects observed here at retrieval may reflect consequences of the age-related differences at encoding. If older adults encoded a less detailed representation regarding the spatial relations between objects compared to younger adults, or if younger and older adults fundamentally differed in the type or quality of the information that was encoded, then the retrieval strategy itself, and the neural regions that govern that retrieval process, may differ due to the change in the nature of the information that was maintained. However, regardless of the nature of the information that was encoded and subsequently retrieved by older adults, they nonetheless engaged similar oscillatory mechanisms as younger adults to maintain the information online during the delay phase. This work adds to the literature regarding the disproportionate decline in relational binding memory impairment in aging [1,2], and points to changes in underlying neural mechanisms that support encoding and retrieval prior to changes in hippocampal volume and behaviour. Oscillatory measures may then be a more sensitive marker of cognitive integrity, or provide an early screen for impending cognitive changes, than memory performance and/or structural integrity.

## Supporting information

S1 Fig Stimulus-locked test phase brain-behaviour differences for accuracy across groups when theta is defined as 4-7Hz. (A, left). The presented contrast shows that the relationship is reliable for both age groups (confidence bounds do not cross zero). (A, right) The scatterplot recapitulates the effects by showing the distribution across individuals across groups with no obvious outliers present. (B) Distribution of virtual channels that positively express the contrast is shown for the three frequency bands of interest. Both age groups showed theta (4-7Hz), alpha, and beta power increases that predicted higher task accuracy.
(TIF)

S2 Fig Oscillatory changes during the test phase (pre-stimulus vs. test phase). Error bars signify confidence bounds (95%) obtained from the bootstrap distribution. Distribution of virtual channels that positively (red) and negatively (blue) express the contrast are shown on the bottom. Older adults showed an occipital theta increase, and widespread power decreases across all frequencies.
(TIF)

S3 Fig Response-locked oscillatory power changes (pre-stimulus vs. test phase) across groups were captured by a single latent variable. Oscillatory responses were examined for the test phase as restricted to the period prior to the response versus the pre-stimulus period. Error bars signify confidence bounds (95%) obtained from the bootstrap distribution. Distribution of virtual channels that positively (red) and negatively (blue) express the contrast are shown on the right for three frequency bands of interest (theta, alpha, and beta). Older adults express the pattern more reliably than younger adults across all frequency ranges.
(TIF)

S4 Fig Response-locked brain-behaviour differences across groups (pre-stimulus vs. pre-response). (A, left) Contrast shows group differences that are reliable for older adults only (A, right) The scatterplot shows the distribution of individuals from each age group. (B) Distribution of virtual channels that negatively express the contrast are shown for the theta and beta frequency ranges. Older adults showed a power decrease in that predicted longer response times.
(TIF)
