# Supplementary figures and images for: Age-related changes to oscillatory dynamics during maintenance and retrieval in a relational memory task
Source: PLoS One. 2019 Feb 7;14(2):e0211851. doi: 10.1371/journal.pone.0211851 (PMC6366750; doi:10.1371/journal.pone.0211851)

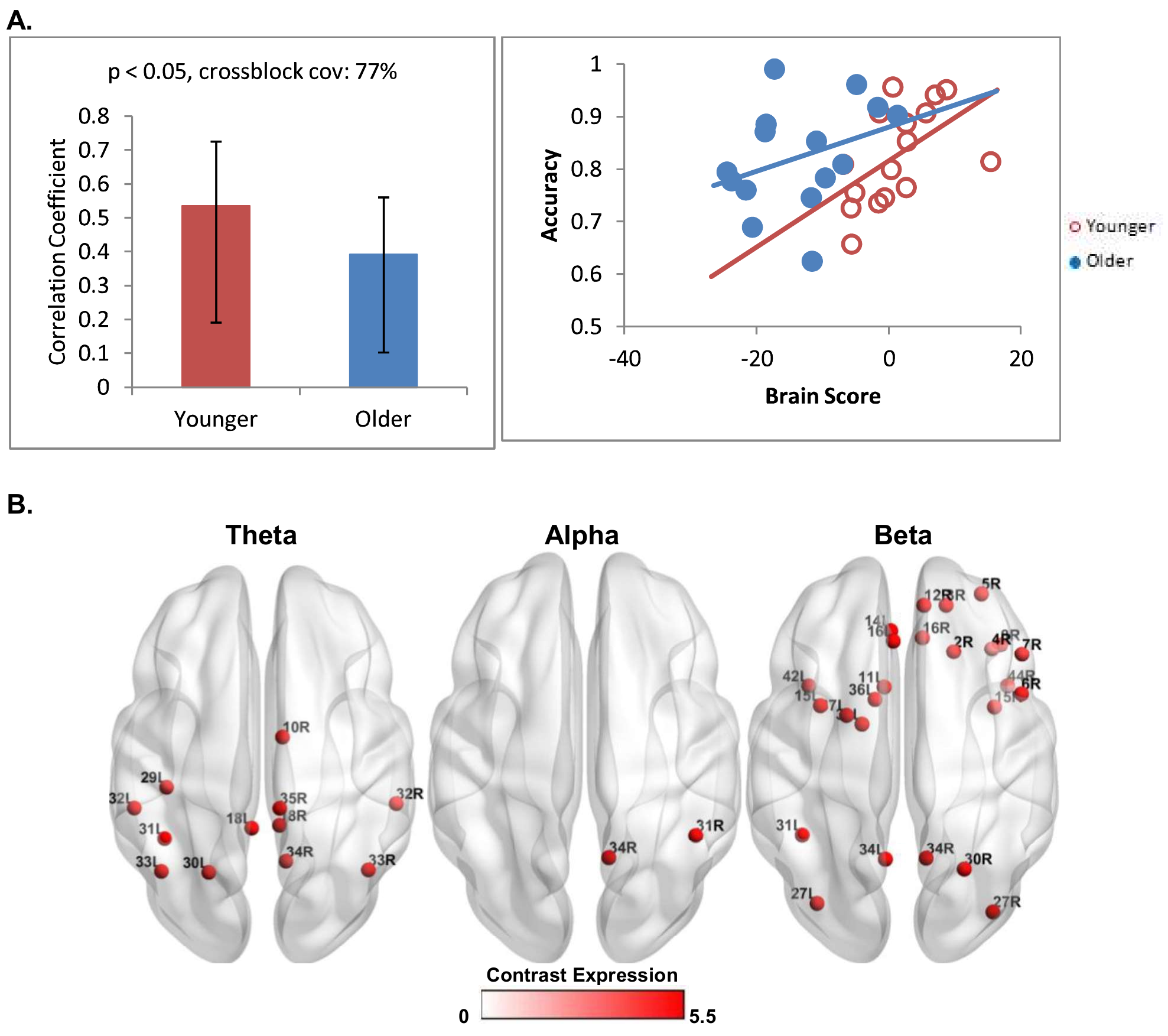

Supplement: S1 Fig — (A, left). The presented contrast shows that the relationship is reliable for both age groups (confidence bounds do not cross zero). (A, right) The scatterplot recapitulates the effects by showing the distribution across individuals across groups with no obvious outliers present. (B) Distribution of virtual channels that positively express the contrast is shown for the three frequency bands of interest. Both age groups showed theta (4-7Hz), alpha, and beta power increases that predicted higher task accuracy. (TIF) [file pone.0211851.s001.tif]

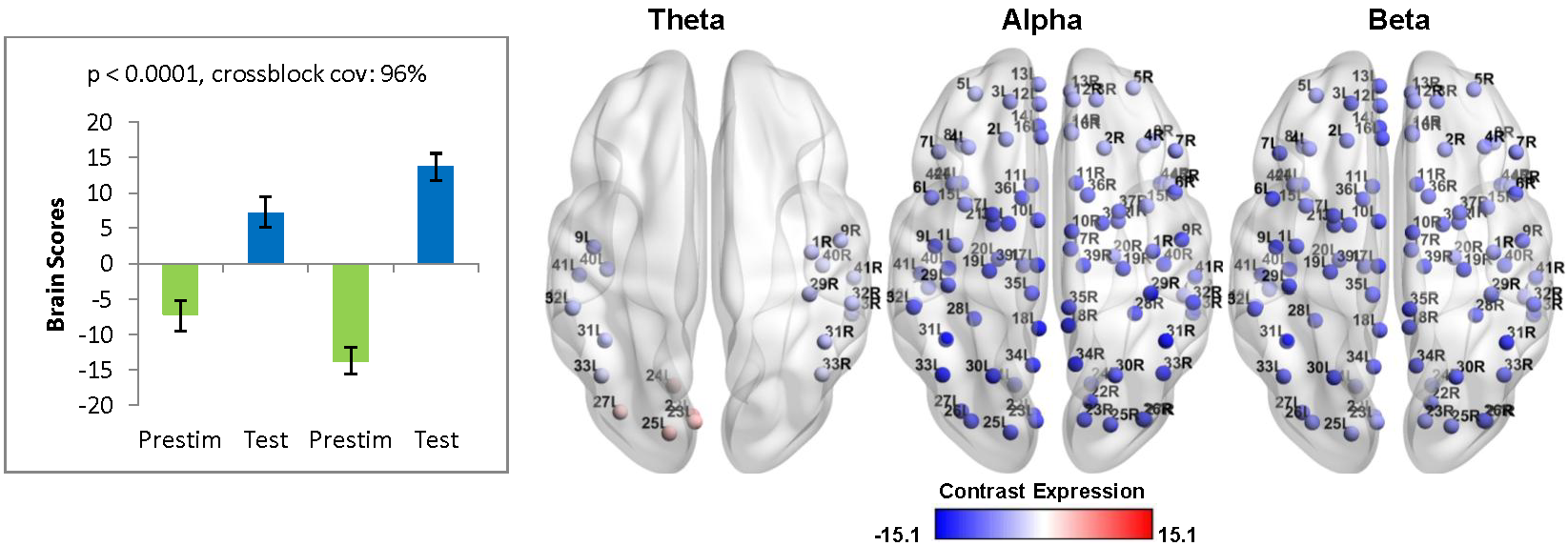

Supplement: S2 Fig — Error bars signify confidence bounds (95%) obtained from the bootstrap distribution. Distribution of virtual channels that positively (red) and negatively (blue) express the contrast are shown on the bottom. Older adults showed an occipital theta increase, and widespread power decreases across all frequencies. (TIF) [file pone.0211851.s002.tif]

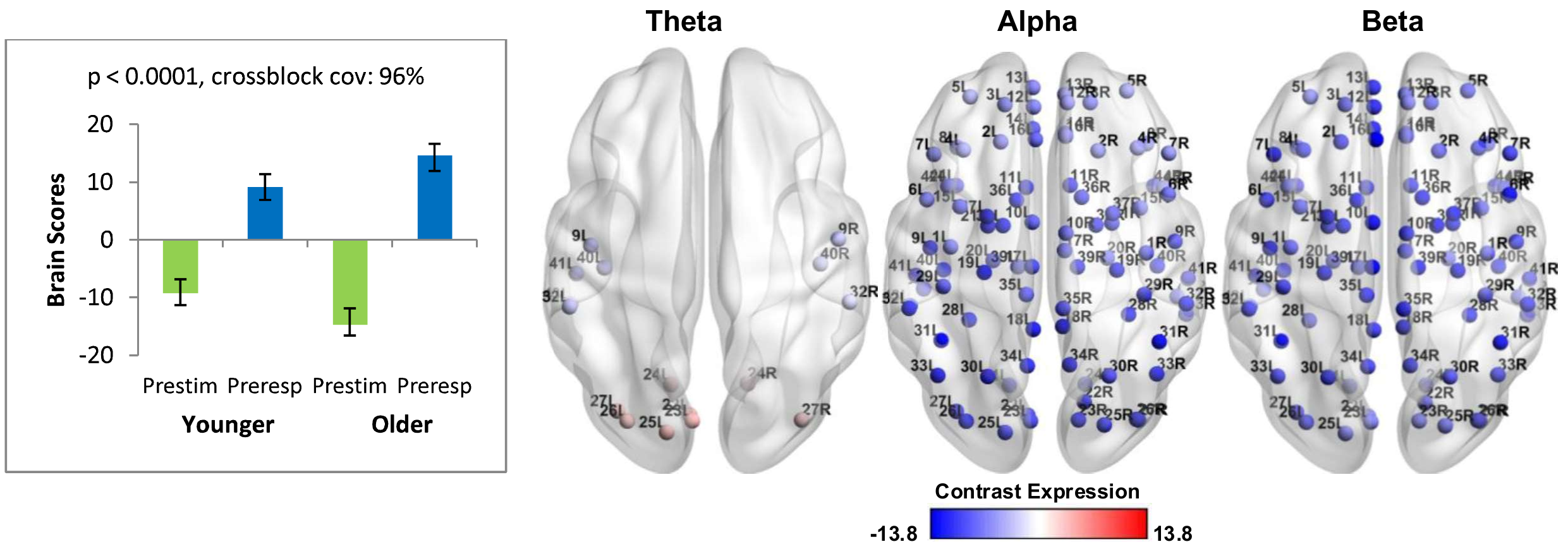

Supplement: S3 Fig — Oscillatory responses were examined for the test phase as restricted to the period prior to the response versus the pre-stimulus period. Error bars signify confidence bounds (95%) obtained from the bootstrap distribution. Distribution of virtual channels that positively (red) and negatively (blue) express the contrast are shown on the right for three frequency bands of interest (theta, alpha, and beta). Older adults express the pattern more reliably than younger adults across all frequency ranges. (TIF) [file pone.0211851.s003.tif]

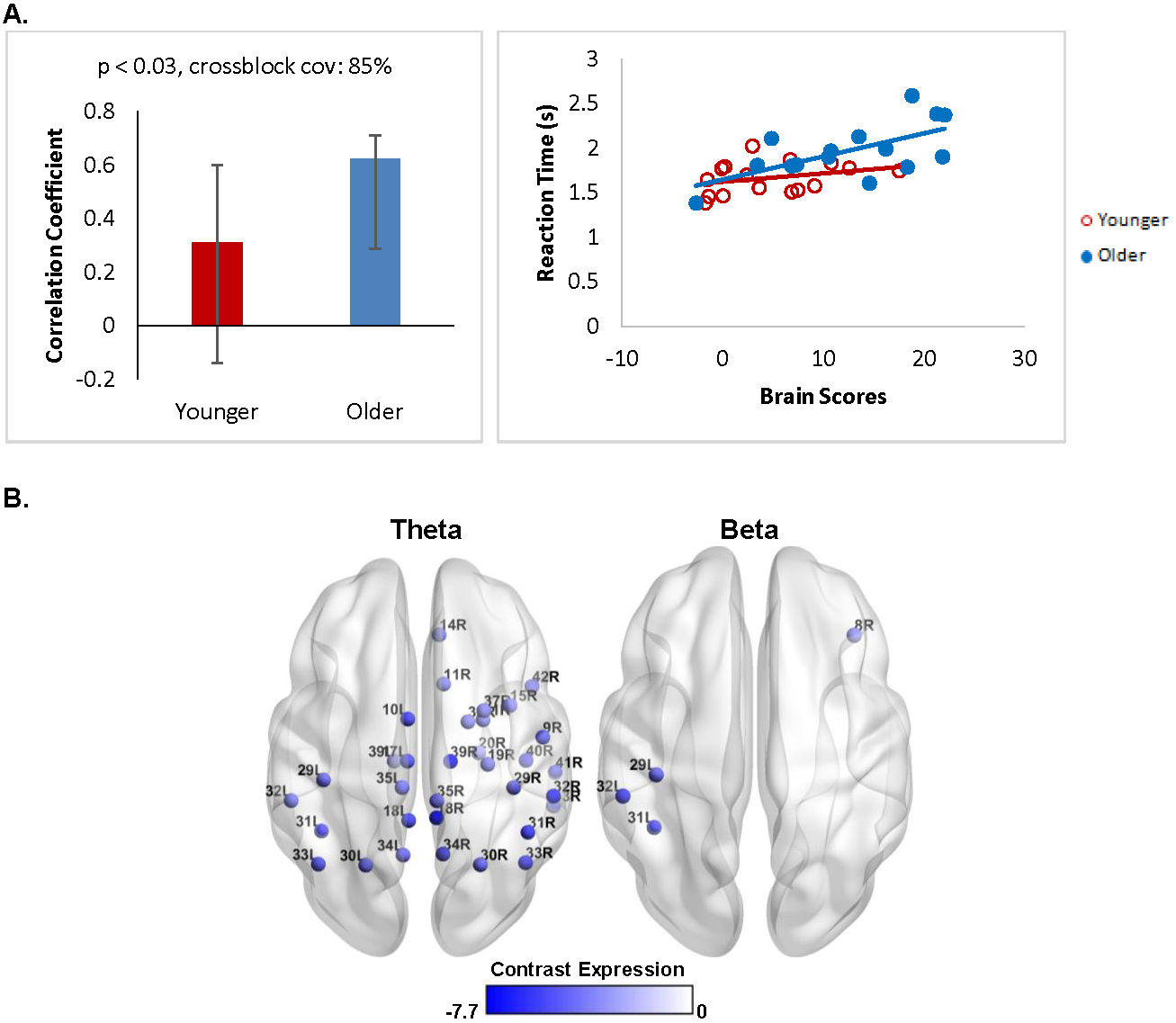

Supplement: S4 Fig — (A, left) Contrast shows group differences that are reliable for older adults only (A, right) The scatterplot shows the distribution of individuals from each age group. (B) Distribution of virtual channels that negatively express the contrast are shown for the theta and beta frequency ranges. Older adults showed a power decrease in that predicted longer response times. (TIF) [file pone.0211851.s004.tif]
